# Acute exposure to diisopropylfluorophosphate in mice results in persistent cognitive deficits and alterations in senescence markers in the brain

**DOI:** 10.3389/fnins.2024.1498350

**Published:** 2024-11-07

**Authors:** Alvin V. Terry, Wayne D. Beck, Victoria Zona, Yutaka Itokazu, Ashutosh Tripathi, Amit Kumar Madeshiya, Anilkumar Pillai

**Affiliations:** ^1^Department of Pharmacology and Toxicology, Medical College of Georgia, Augusta, Georgia; ^2^Small Animal Behavior Core, Medical College of Georgia, Augusta, Georgia; ^3^Department of Psychiatry and Behavioral Sciences, McGovern Medical School, The University of Texas Health Science Center at Houston, Houston, TX, United States; ^4^Medical Research Service, Charlie Norwood VA Medical Center, Augusta, Georgia

**Keywords:** organophosphate, nerve agent, memory, acetylcholine, cognition, gulf war illness, pesticide, aging

## Abstract

Organophosphates (OPs) are found in hundreds of important products used worldwide; however, they have been associated with adverse long-term health consequences ranging from neurodevelopmental deficits to age-related neurological diseases. OP exposure has also been implicated in Gulf War Illness; a cluster of medically unexplained chronic symptoms estimated to affect 25–32% of veterans of the Persian Gulf war in 1991. The development of multiple types of chronic illnesses in these veterans at an early age compared to the general population has led to the suggestion that they are experiencing signs of premature or accelerated aging. The process of cellular senescence and the development of the senescence-associated secretory phenotype (SASP) is believed to lead to chronic inflammation, chronic illnesses, as well as accelerated biological aging, and a role of environmental exposures in these processes has been suggested, but not extensively studied to date. In the studies described here, we evaluated the persistent effects of a single (acute) exposure of a representative nerve agent OP, diisopropylfluorophosphate (DFP) 4.0 mg/kg on cognitive function, noncognitive behaviors, cellular senescence markers and proinflammatory cytokines in the mouse brain. The results indicated modest, but persistent DFP-related impairments in spatial learning and working memory, but not contextual or cued fear conditioning. DFP exposure was also not associated with negative effects on weight or impairments of the various noncognitive (e.g., motor function or exploratory activity) behavioral assessments. Both histology and quantitative PCR experiments indicated that DFP was associated with persistent alterations in several senescence markers and proinflammatory cytokines in brain regions that are relevant to the performance of the memory-related tasks (e.g., hippocampus, prefrontal cortex). The results thus suggest that single acute exposures to OPs like DFP can lead to persistent impairments in specific domains of cognition that may be related to alterations in cellular senescence and inflammaging in the brain.

## 1 Introduction

The diverse chemicals known as the organophosphates (OPs) are found in hundreds of products used worldwide including pesticides, defoliants, fire retardants, industrial solvents, lubricants, plasticizers, fuel additives, and chemical weapons/nerve agents (see reviews, Soltaninejad and Shadnia, 2014; Costa, 2018). The extensive use of OPs especially as pesticides has been a public health concern for decades given their epidemiological associations with adverse long-term health consequences (Rohlman et al., 2011) which can range from neurodevelopmental deficits to age-related neurological diseases such as Alzheimer’s disease (AD) and Parkinson’s disease (PD) (see reviews, Zaganas et al., 2013; Sánchez-Santed et al., 2016; Yan et al., 2016; Mostafalou and Abdollahi, 2018). Acute high dose OP exposure from pesticides is also common in suicide attempts which also represents a major global health challenge (Mew et al., 2017; Huang et al., 2020). The heavy use of OP pesticides during the 1990–1991 Persian Gulf War (Operation Desert Shield and Operation Desert Storm) including azamethiphos, chlorpyrifos, dichlorvos, diazinon, and malathion, has also been suggested as one of the causal factors contributing to chronic symptoms of Gulf War Illness (GWI) (U.S. Government Printing Office, 2008; see also review, Ribeiro and Deshpande, 2021). These symptoms can include unexplained fatigue, headaches, respiratory problems, musculoskeletal pain, gastrointestinal distress, skin rashes, and a variety of neurological and neuropsychiatric problems including cognitive impairment (Sullivan et al., 2018). There is also significant evidence from atmospheric modeling and satellite imaging data to suggest that up to 100,000 Gulf War soldiers may have been exposed to low levels of the nerve agent OPs sarin and cyclosarin following the destruction of an Iraqi munitions storage complex at Khamisiyah, Iraq, in March 1991 (US Department of Defense, 2002a, 2002b; U.S. Government Printing Office, 2008; White et al., 2016). Interestingly, the development of multiple types of chronic illnesses in Gulf War veterans at an early age compared to the general population has led to the suggestion that these veterans are experiencing signs of premature or accelerated aging (Zundel et al., 2019).

The threat from intentional OP-nerve agent poisonings by rogue governments and terrorists, is also an ever-present concern. Over the last 40 years, there have been multiple (well-documented) cases where OP nerve agents were used against military soldiers and/or civilians. Examples include Iraqi military attacks on Iranian soldiers and Kurdish civilians in the 1980s, the Tokyo Sarin attacks by the domestic terrorist group Aum Shinrikyo in 1995, multiple military attacks on civilians in Syria with Sarin between 2013 and 2018, the use of the OP VX in the assassination of the North Korean dictator’s half-brother in Malaysia in 2017, and the attempted assassinations of opponents of the Russian government with the OP Novichok (e.g., the Skripals in 2018 in Britain and Alexei Navalny in Russia in 2020) (see review, Naughton and Terry, 2018; Steindl et al., 2021). Compared to OP pesticide exposures, there is less published literature available on the long-term effects of the nerve agent exposures described above, however, persistent neurologic effects (including psychomotor impairments and cognitive deficits) in the victims of the aforementioned Tokyo Sarin attacks and the Iraqi military attacks have been reported (Nishiwaki et al., 2001; Miyaki et al., 2005; Loh et al., 2010; Talabani et al., 2018).

Regarding the potential for OP exposures to accelerate the biological aging process mentioned above, there are a few studies that directly link OP exposures to cellular senescence which is defined as a complex stress response resulting in a permanent arrest of cell proliferation and the development of the senescence-associated secretory phenotype (SASP) (Acosta et al., 2008; Coppé et al., 2008; Coppé et al., 2010; Kuilman et al., 2008). The SASP is characterized by the cellular release of multiple cytokines, chemokines, growth factors, proteases, and lipids which promotes inflammation and the immune-mediated suppression of tumor growth and metastasis, limits fibrosis, and facilitates wound healing and tissue regeneration (Lopes-Paciencia et al., 2019). The SASP thus contributes to the preservation or restoration of tissue homeostasis, and it is typically transient in younger, healthy tissues (Tchkonia et al., 2013; Neves et al., 2015; Basisty et al., 2020). However, a growing body of evidence suggests that a chronic SASP can lead to inflammaging and, consequently, the development of age-related disease pathology.

As one example of OP-related cellular senescence, the OP agricultural pesticide phosalone increased senescence in rat embryonic fibroblast cells in culture, an effect that was attributed to the elevation of oxidative stress factors, the expression of aging-related genes, inflammatory cytokines, cell cycle arrest, apoptosis and necrosis and shortening of telomerase (Baeeri et al., 2017). In another study, the OP-based flame retardant, Tris (1-chloro-2-propyl) phosphate (TCPP) was associated with DNA damage and cellular senescence as well as decreases in the viability of cultured human skin keratinocytes. These effects were attributed to elevations in SASP proinflammatory cytokines, the upregulation of mRNA expression of p53 and p21 tumor-suppressor genes and decreases in the expression of the cell cycle regulator, cyclin D1 (Liu et al., 2022). To our knowledge there has been only one study conducted *in vivo* in a mammalian model to date to evaluate the effects of OPs on cellular senescence. This study was conducted in rats where diisopropylfluorophosphate (DFP) + OP antidotal agents, atropine and 2-pralidoxime (25 mg/kg, i.m.) was associated with both a time dependent and neuron-specific appearance of senescence markers after drug exposure (Tsai et al., 2024, see further details of this study in the Discussion). In the studies described in this report, we evaluated the persistent effects of a single exposure to DFP alone on cognitive function, noncognitive behaviors, cellular senescence markers, and proinflammatory cytokines in the mouse brain. DFP is a prototypical alkylphosphate OP that possesses a great deal of structural homology with highly toxic nerve agents such as sarin and soman, but is less potent and lethal (Hobbiger, 1972).

## 2 Methods

All animal procedures employed during this study were reviewed and approved by the Institutional Animal Care and Use Committee and are consistent with AAALAC guidelines. Measures were taken to minimize pain or discomfort in accordance with the Guide for the Care and Use of Laboratory Animals, 11th edition, National Research Council, 2011. Significant efforts were also made to minimize the total number of animals used while maintaining statistically valid group numbers.

### 2.1 Test subjects

Three-month-old, male C57BL/6 mice were obtained from Envigo, Indianapolis, IN and housed at the Small Animal Behavior Core (SABC) at Augusta University in a temperature-controlled room (25°C), maintained on a 12-h light/dark cycle with free access to food (Teklad Rodent Diet) and water throughout the study. Table 1 provides the details for all study cohorts, the numbers of animals tested per group, and the experiments conducted in each group at different time points.

**Table 1:** Mouse testing protocol.

Cohort	*N*	Treatment	Procedure/testing days
1*	11-17	VEH	Water Maze (6–13), Y-Maze (14),
2*	12–17	DFP	Hanging Wire (15), grip strength (16)
			Rotarod (17–18), Phenotyper (20–23)
			Visual Reaching Response (29), sacrifice (35)
			qtPCR (post day 35)
3	10	VEH	Fear Conditioning (22–24), sacrifice (35)
4	14	DFP	
5	6	VEH	RBC and Brain AChE (day 0, I hr. post DFP)
6	6	DFP	
7	5	VEH	RBC and Brain AChE (day 13 post DFP)
8	6	DFP	
9	5	VEH	RBC and Brain AChE (day 35 post DFP)
10	6	DFP	
11	3	VEH	Histology (7)
12	3	DFP	

VEH, Vehicle; DFP, Diisopropylfluorophosphate. *Indicates the minimal to maximal number of animals within the cohort that were included in the procedures indicated. The *N* for the individual number of animals in a particular group in these tasks are provided in the Fig legends and Table 2.

### 2.2 Diisopropylfluorophosphate administration and observational studies

In this report, we evaluated a 4.0 mg/kg dose of DFP which can produce seizure activity and cholinergic signs in mice but is also associated with prolonged neuroinflammation in the brain as indicated by elevations in of inflammatory cytokines (O’Callaghan et al., 2015; Locker et al., 2017). The published LD50s for DFP most relevant to these studies (i.e., DFP administered by i.p. injection specifically in C57BL/6 mice) are found in Smolen et al. (1985) and are 6.8 mg/kg and 6.33 mg/kg for male and female mice, respectively. In the studies described here, we found that less than 20% of the mice administered this dose died. Each experimental group received vehicle (0.9% Blood Bank Saline, Fisher Scientific) or DFP, CAS 55–91-4 (Sigma Aldrich D0879-1G Lot: 126 K1306, St. Louis, MO) 4.0 mg/kg dissolved in vehicle by intraperitoneal injection in a volume of 10x body weight. Individual mice were monitored (in their home cages for a period of approximately 5 min each day) for visible cholinergic signs (diarrhea, excessive salivation or lacrimation, respiratory difficulties, muscle fasciculations) or other signs of distress throughout the study. Test subjects were weighed every 3–4 days throughout the 35-day study.

### 2.3 Acetylcholinesterase activity

Acetylcholinesterase (AChE) activity was assessed in red blood cells (RBCs) and brain using the method of Ellman et al. (1961) with modifications to accommodate a 96-well microplate format at 25°C as we have described previously (see Terry et al., 2007; Naughton et al., 2018). Blood and brains were collected in separate sets of animals in parallel with the animals in the behavioral and histology studies (see Table 1) at three time points, (1) 1 h after the acute injection of DFP, (2) 13 days after the DFP injection, and 35 days after the DFP injection. At the end of the respective time periods, subjects were anesthetized with isoflurane and blood was taken transcardially and brains were removed. Red blood cells were collected in Potassium/EDTA Microtainer™ Capillary Blood Collector (BD®, Franklin Lakes, NJ) and spun per manufactures instructions to separate RBC’s and plasma. Plasma was aspirated and a 1:50 dilution in 0.9% (w/v) blood bank saline with 1.0% Triton X-100 (Sigma, St. Louis, MO) was made. RBC’s were frozen and stored at −80°C until the Ellman assay was performed. Brains were extracted, rinsed with phosphate-buffered saline (PBS, pH 7.4) and stored at −80°C until use. Brain tissue was homogenized in ice-cold PBS (wt/vol: 1 g wet brain tissue/4 mL PBS) using a motor driven glass-Teflon tissue grinder. Brain and RBC total protein concentration was measured using a Micro BCA Protein Assay Kit (ThermoFisher Scientific Inc., Rockford, IL, United States) according to manufacturer’s instructions. Brain homogenates (20–50 μg protein/μl) and RBC’s (7–12 μg protein/μl) were assayed in duplicate for AChE using 0.48 mM acetylthiocholine (substrate) and 0.52 mM dithiobisnitrobenzoic acid diluted in 1.0 mM sodium phosphate buffer (see Terry et al., 2007). The reaction mixture for RBC’s and brain included 0.097 mM ISO-OMPA (Sigma, St. Louis, MO) and was pre-incubated with the samples 5 min prior to substrate addition for butyrylcholinesterase inhibition. The formation of reaction product (yellow color) was monitored by measuring absorbance values (in optical density) at 412 nm every 2 min for 16 min Cytation 3 microplate reader (Agilent Technologies). The cholinesterase-mediated reaction rate (moles/L per min) was calculated by dividing the change in absorbance per min by 13,600 (Ellman et al., 1961). We expressed the results both as AChE enzyme activity (nmol of the substrate acetylthiocholine hydrolyzed per min per mg of protein) and as a percentage of the mean of the vehicle-treated subjects.

### 2.4 Water maze tests

#### 2.4.1 Test apparatus

The water maze consisted of a circular pool (160 cm in diameter, 60.9 cm in depth) filled to within 15 cm of the rim with water (22°C) made opaque by the addition of nontoxic white Soft Gel Paste™ Food Color (AmeriColor Corp, Placentia, CA). A circular escape platform (Plexiglass; 15.24 cm in diameter) was located 2 cm below the water’s surface in a constant position in one of four imaginary quadrants of the pool. The animal could use only distal, extramaze visual cues (posters hung on the walls, 3-D objects in the testing room, and black colored curtains surrounding parts of the maze room) to locate the submerged platform. Latencies and distances swam to locate the hidden platform during the training and probe trials (see procedures below) were recorded and analyzed using a computer-based video tracking system (Noldus Ethovision XT-14).

#### 2.4.2 Hidden platform task

The animals were given 15 trials over 5 consecutive days (3 trials per day; 20 min inter-trial interval) with the platform submerged. Trial duration was set at a maximum of 90 s; if the platform was not located, the animal was gently guided to the platform and required to remain there for 20 s. The circular pool was divided into 8 equally divided sections which served as start positions; the start position associated with the hidden platform location was not used. Start positions were randomized within and across subjects over the 5 days of training. Animals were placed into the pool facing the sidewall on each trial.

#### 2.4.3 Probe trial

After the last training trial, a 30 s probe trial was performed where the platform was removed. Spatial reference memory was assessed by measuring the latency to the first entry into the annulus-40 defined as a 40 cm diameter target zone centered around the previous hidden platform location. In addition, the percentage time spent in the target quadrant (representing 25% of the total pool area), was also assessed.

### 2.5 Y-maze spontaneous alternation test

#### 2.5.1 Apparatus and scoring methods

The Y-maze is opaque, white-cast acrylic with three arms (each = 30.5 cm L x 7.6 cm W x 12.7 cm H) joined at one end to form a ‘Y’ shape with a common central zone. Both extramaze and intramaze, cues were visible to use as spatial landmarks. The apparatus was cleaned with 50% ethanol between trials. All trials were recorded using a computer-based video tracking system Noldus EthoVision XL Version 13, with the manual scoring option to score the sequence that each mouse entered arms over the testing period. Other outcome measures were recorded including the total distance traveled, and total number of arm entries.

#### 2.5.2 Spontaneous alternation assessment

Each trial was initiated by releasing a mouse into the center zone of the Y-Maze for a 10-min free exploration period. Subsequently, the first two trials were omitted from the data analysis and the total number of alternations (entries into three different arms consecutively) and were counted. The percentage alternation was calculated, as follows: (number of alternations)/(total possible alternations) × 100%.

### 2.6 Contextual and cued fear conditioning

#### 2.6.1 Shock threshold

Prior to the DFP studies, a sequence of single foot shocks was delivered to male C57BL/6 mice placed on the same electrified grid used for fear conditioning (see below) in order to assess the shock (i.e., sensory perception) threshold. Initially, a 0.1-mV shock was delivered for 1 s, and the animals’ behavior was evaluated for flinching, jumping, and vocalization. At 30-s intervals the shock intensity was increased by 0.1 mA up to 0.8 mA and then returned to 0 mA in 0.1-mA increments at 30-s intervals. Threshold to vocalization, flinching, and then jumping was quantified for each animal by averaging the shock intensity at which each animal manifested a behavioral response to the foot shock. This average was determined (0.75 mA, 2 s) and used in the subsequent DFP experiments.

#### 2.6.2 Training procedure

Separate groups of mice were subsequently evaluated for DFP-related effects on contextual and cued fear conditioning using the mA shock levels determined in the experiments described above. A three-pairing method of auditory cue and mild foot shock was conducted during a 6.5 min test session. Animals were allowed to freely explore the apparatus (MED-VFC-NIR-M, Med Associates, St Albans, VT) for 3 min (180 s-baseline) followed by a tone (CS, 20 s, 2 kHz, 85 dB). After termination of the tone, a foot shock (US, 0.75 mA, 2 s) was delivered through the stainless-steel grid floor. Mice received three foot shocks with an intertrial interval of 60 s. Subjects were removed from the fear conditioning chamber 30 s after shock termination and returned to their home cages.

#### 2.6.3 Contextual fear

In the contextual fear conditioning version of the task (conducted 24 h after training sessions), the mice were placed back into the original training context for 8 min, during which no foot shock was delivered.

#### 2.6.4 Testing-cued fear

In the auditory-cued fear-conditioning version of the test (conducted 48 h after training sessions) animals were placed into a novel context (same chambers, but with different walls, floor, and background odor), and after a 3 min baseline period, they were continuously re-exposed to the tone (same characteristics as the conditioning stimulus) for 5 min, but in the absence of shocks.

#### 2.6.5 Data analysis

The Med-Associates Video Freeze® Software program was used for scoring individual test subjects from the recorded videos. Freezing was defined as behavioral immobility except for movement needed for respiration.

### 2.7 Visual reaching response test

The general visual capability of each mouse was determined by the visual reaching response, according to a modified version of the method described by Landmann et al., 2019. Briefly, each mouse was initially allowed to explore the experimental setup (top shelf of a stainless-steel service cart with 2 cm lip) for 30 s prior to the visual test. Subsequently, each mouse was held by its tail and elevated to a height of approximately 20 cm above the surface of the cart and slowly and steadily lowered to the surface. As described by Landmann et al., 2019, mice with well-developed vision stretch their forepaws as soon as they recognize the surface of the experimental arena, prior to the landing. Each mouse was elevated five times with an intertrial interval of 10 s. The observed behavior was scored as follows: 0 = no leg move, 1 = legs stretched, 2 = legs stretched early (>1.5 cm from the surface of the cart).

### 2.8 Grip strength test

A modified version of the forelimb grip strength test (see Takeshita et al., 2017) was used for these experiments. Strength was measured with a digital grip strength meter (Chatillon Digital Force Gage DFIS 2, C.S.C Force Measurement, Inc.) by holding the mouse by the nape of the neck and by the base of the tail. The forelimbs were placed on the tension bar and the mouse was pulled back gently until it released the bar. Each animal was assessed three times and mean grip strength (measured in grams of resistance ± SEM) was calculated. The average grip strength was then normalized to the weight of the individual test subject (in grams).

### 2.9 Hanging wire test

A modification of the four-limb hanging wire test of Van Putten et al., 2010 was used in these experiments. An acrylic box (40.6 × 40.6 × 30.5 cm) with corn cob bedding covering the floor served as the main test apparatus. Mice were placed in the center of a wire mesh cover (12 mm x 12 mm mesh opening size) which was then inverted and placed on the top of the box. Mice were free to move around on the mesh and 4 total trials were conducted. The first trial was used for training and if the test subject fell from the mesh, it was placed back on the mesh for additional training and the initial hang time was recorded, although this value was not considered in the final analysis. For the 3 remaining trials, the hang time was recorded, and the trial ended when the subject fell off the mesh or it reached 5 min. The mean of the 3 hang times per subject was used in the group comparisons.

### 2.10 Rotarod test

Motor coordination, balance, motor learning, and endurance were evaluated with an accelerating rotarod method (Rotor-Rod System, San Diego Instruments, San Diego, CA). For the training day, each mouse was given 3 trials consisting of the bar accelerating from 0 to 10 rpms over a 1-min period for a duration of 5 min. The test was performed 24 h following the training and mice were given a “refresher” trial the same as training, followed by 3 trials where the bar accelerated gradually from 0 to 23 rpms over 2 min for a duration of 5 min. The amount of time elapsed before each subject fell from the rod onto a padded surface was recorded. On both days, each test subject had a 20 min intertrial interval.

### 2.11 Phenotyper home cage observations

Open field locomotor and exploratory activity, drinking behavior, voluntary exercise, and anxiety-like behaviors were assessed in Noldus Phenotyper Home Cage Systems for mice equipped with lickometers, voluntary running wheels, infrared translucent shelters, and a feeding station. The cages (30 × 30 × 30 cm) are made of transparent plexiglass walls with an opaque plexiglass floor which was covered with bedding. Each cage lid is equipped with infrared LEDs and an infrared-sensitive camera for video tracking. EthoVision XT 16 software (Noldus Information Technology, The Netherlands) was used for video tracking trial control and data analyses and export. Each mouse was placed in one of eight Phenotyper Home Cages for one 18-h habituation session followed the next day by one 18-h test session. Only the test session was recorded and analyzed. The outcome measures recorded for the 18 h test session (totals), were the distance traveled (cm), number of licks on the lickometer, running wheel revolutions, time on the running wheel (sec), number of shelter entries, and time in the shelter (sec).

### 2.12 Immunohistochemistry staining

Seven days after DFP exposure a separate group of mice were anesthetized with isoflurane using an open-drop method and transcardially perfused with phosphate-buffered saline (PBS, pH 7.4) and 4% paraformaldehyde (PFA) in PBS. After perfusion, the brains were removed and post-fixed in 4% PFA at 4°C for 24 h. The blocks were then equilibrated in sucrose (30% in PBS). Cryosections were cut at a thickness of 30 μm. Sections from prefrontal cortex (PFC) were blocked and permeabilized with 1% Tween 20 in 1.0% bovine serum albumin (BSA) in PBS, at room temperature for 30 min, then washed with PBS before immunohistochemical staining. Sections were incubated in the following primary antibodies 37°C for 1 h: rabbit anti-p21 (RRID:AB_823586; Cell Signaling Technology, #2947S, 1:500), mouse anti-NeuN (RRID:AB_2298772; Sigma-Aldrich, #MAB377, 1:250), mouse anti-Glial Fibrillary Acidic Protein (GFAP) (RRID:AB_477010; Sigma-Aldrich, #G3893, 1:250), goat anti-Iba1 (RRID:AB_ 2,224,402; Abcam, #ab5076, 1:50). After three washes with PBS, the samples were incubated with appropriate secondary antibodies coupled to Alexa488 or Alexa 568 (Invitrogen) at a dilution of 1:1,000 at room temperature for 2 h. Nucleus counterstaining was performed with 1 μg/mL 40,6-diamidino-2-phenylindole (DAPI) (Thermo Fisher Scientific, #D1306) for 30 min. After washing with PBS 3 times, the sections were mounted with Vectashield mountain medium with DAPI (Vector Laboratory, #H-1000).

### 2.13 Fluorescent imaging and cell counting

Fluorescent images were acquired using a Cytation 3 cell imaging multi-mode microplate reader (Agilent Technologies) equipped with a 60x objective with identical acquisition settings. Gen5 software was used for initial image acquisition of fluorescent signals. Confocal images were acquired using a Zeiss LSM 780 with a 40x oil objective (Zeiss, Land Baden-Württemberg, Germany) with identical acquisition settings. Zen software was used for initial image acquisition of the fluorescent signals. Quantitative analyses of digital images were performed using Fiji (NIH, Bethesda, MD, United States) and merged using Photoshop (Adobe, San Jose, CA, United States). p21 positive cells in the prefrontal cortex (PFC) were counted with the DAPI-staining for quantification (n = 3 mice, five sections per each mouse). Double-positive cells were identified and counted when their staining overlapped. Immunofluorescence higher than background and specific staining pattern/localization were considered to determine positive/negative for each staining when cells were counted using Fiji image analysis. To calculate the total number of marker protein-positive cells, five sections per animal were analyzed. To calculate the total number of marker-double-positive cells, at least 1,000 cells per group were analyzed. Blinding procedures and the randomized field approach for images were performed to acquire unbiased results.

### 2.14 Molecular (q-RT-PCR) studies

In these experiments, at the end of a 35-day washout period after DFP exposure, we measured the mRNAs of two of the most commonly evaluated senescence markers p21 (CDKN1a) and p16 (CDKN2a), members of the proinflammatory CXCL chemokine family cxcl1, cxcl2, and ccl8, matrix metalloproteinases, MMP3 and MMP12, known to be effectors of inflammatory processes, timp1, an endogenous inhibitor of MMPs, but also known to have proinflammatory roles beyond MMP inhibition, and interleukins, il1a, il1b and il16, potent pro-inflammatory cytokines. Total RNA from the PFC and hippocampus (*n* = 6) samples was isolated using a commercially available kit (SV RNA Isolation, Promega, Madison, WI, United States). All RNA samples were quantified using a Nanodrop. After the quantification, cDNA was prepared using iScript™ cDNA Synthesis Kit (Bio-Rad CA, United States). Quantitative reverse transcription polymerase chain reaction (q-RT-PCR) was performed on a Master Cycler (Quant Studio-7 Real-Time PCR Systems, Thermo Fisher Scientific, United States) using iTaqTM Universal SYBR® Green Super mix (Bio-Rad, CA, United States). Primers were synthesized by Integrated DNA Technologies. Ct values of genes of interest were normalized to that of housekeeping gene, beta2-microglobulin (B2M). The list of primers used is given in Supplementary Table S1.

### 2.15 Statistical analysis

All data were collated and entered into Microsoft Excel spreadsheets. The data were subsequently imported into SigmaPlot® 11.0 or GraphPad Prism version 10.0 for statistical analyses. Unpaired (two-tailed) *t*-tests and two-factor analysis of variance (ANOVA) were used with repeated measures, followed by the Holm-Sidak post-hoc test. All results were expressed as the mean (± S.E.M.). Differences between means from experimental groups were considered significant at the *p* < 0.05 level. The statistical *F* values and degrees of freedom associated with the ANOVA analyses are provided for the outcome measures when the *p* values were statistically significant (*p* < 0.05).

## 3 Results

### 3.1 Initial observations and weight assessments

Most of the mice administered DFP 4.0 mg/kg exhibited some signs of elevated cholinergic activity (e.g., diarrhea, salivation, decreased exploratory activity, mild muscle fasciculations) which were fully resolved in approximately 24 h As noted in the Methods section above, test subjects were weighed at the beginning of the study and every 3–4 days throughout rest of the 35-day study. The control subjects’ initial average weights (mean ± s.e.m) were 32.2 ± 0.63 grams and the DFP treated subjects’ initial average weights were 31.8 ± 0.67. On the last day of the study (day 35), the control subjects’ average weights were 33.8 ± 0.75 grams and the DFP treated subjects’ average weights were 32.7 ± 0.88. The group weights were not significantly different at any point during the study.

### 3.2 Cholinesterase activity (RBC and brain)

**Figure 1:**
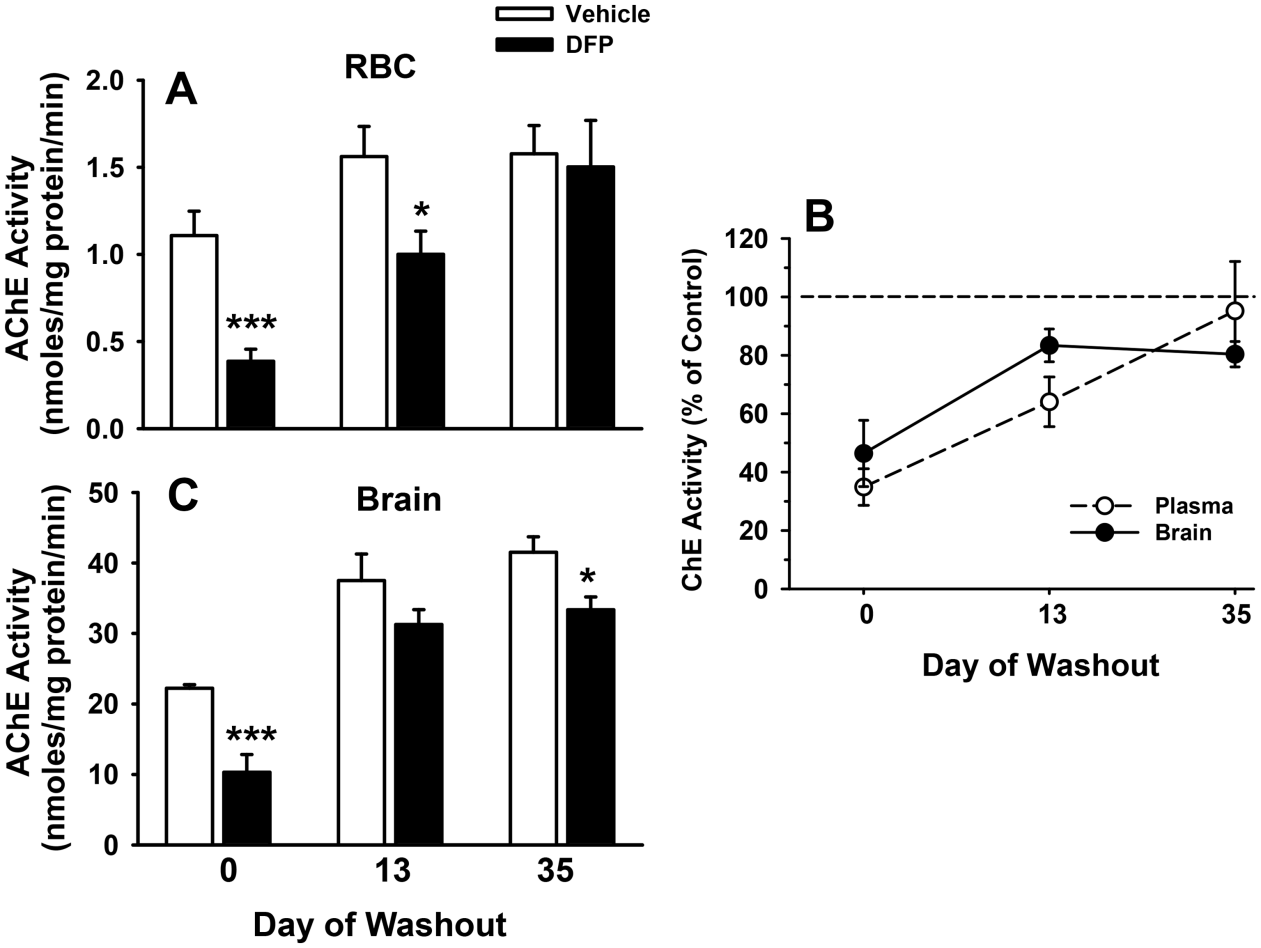
The effects of DFP 4.0 mg/kg on RBC and brain cholinesterase activity in mice at 1 h (day 0), 13 days, and 35 days after exposure. **(A)** AChE activity in RBCs expressed nmoles of acetylthiocholine hydrolyzed per min per mg of protein. **(B)** AChE activity in brain expressed nmoles of acetylthiocholine hydrolyzed per min per mg of protein. **(C)** Data (mean ± SEM) presented as % of vehicle matched control levels. (*N* = 5–6). ***p < 0.001; *p < 0.05, for comparison of DFP to vehicle.

DFP 4.0 mg/kg produced a significant decrease in RBC and brain cholinesterase activity as expected (See Figures 1A–C). Maximal inhibition in RBCs was detected at the initial assessment conducted 1 h after the injection where enzyme activity was reduced to about 35% of control (Figures 1A,C). During the washout period, RBC cholinesterase activity returned in a linear fashion to approximately 64% of control at the 13-day time point and to >95% of control at the 35-day time point. In the brain, maximal cholinesterase inhibition was also detected 1 h after the DFP injection where enzyme activity was reduced to about 46% of control (Figures 1B,C). During the washout periods, cholinesterase activity markedly increased, but did not fully return to control levels (~83% of control at the 13-day and ~ 80% of control at the 35-day time points, respectively).

### 3.3 Water maze studies

#### 3.3.1 Hidden platform test

**Figure 2:**
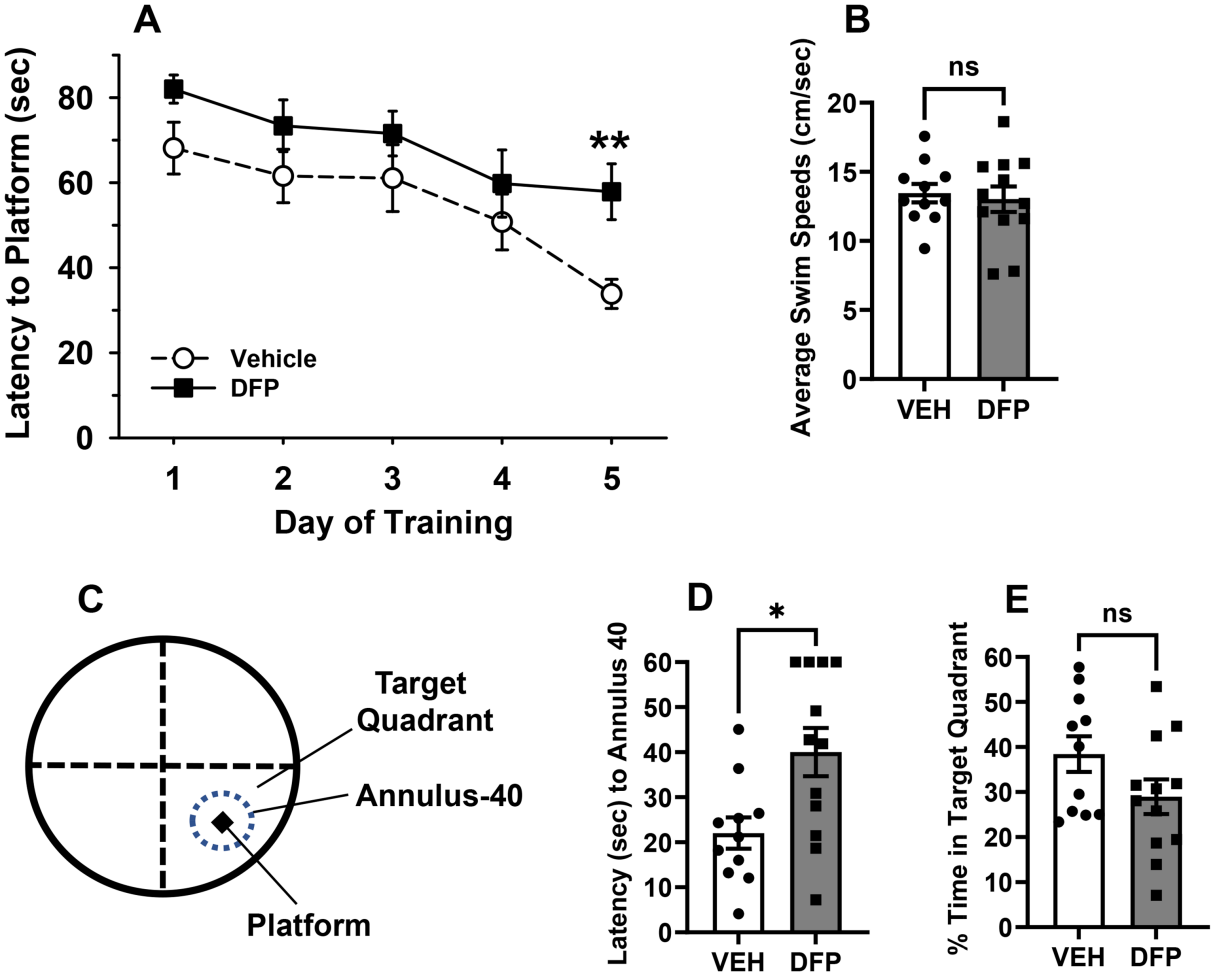
Persistent effects of DFP in mice in the water maze. Beginning 6 days after a single injection of DFP 4.0 mg/kg i.p., young male mice were trained in the water maze 3 trials per day for 5 consecutive days to locate and climb onto a submerged hidden platform. **(A)** mean latency in sec ± SEM for each testing day; **(B)** Average swim speeds in cm/s over the 5 days of hidden platform testing. **(C)** Diagram of the pool with the location of the platform location for probe trials probe trial, with a 40 cm diameter target zone centered around the previous hidden platform location (designated annulus 40) and the larger target quadrant of the previous platform location (25% of the total pool area) **(D)** Latency (sec) to enter the 40 cm annulus ring around the previous platform area in a probe trial conducted after the hidden platform testing **(E)**. Probe trial-% time spent in the quadrant where the platform had been located. **p < 0.01, *p < 0.05, DFP- vs. vehicle effect on performance. Vehicle, *N* = 11; DFP, *N* = 12.

Figure 2A illustrates the efficiency (mean latencies ± SEM) of mice that had been previously exposed (i.e., 6 days earlier) to saline or DFP (4.0 mg/kg, i.p.) to locate a hidden platform in the water maze task over 5 consecutive days of testing. The mice in each experimental group learned to locate the hidden platform with increasing levels of efficiency over the course of the 5 days as evident by the decreasing slope of the acquisition curves. Statistical analyses provided the following results: main effect of group (*F*_1,21_ = 4.8, *p* < 0.04), session (*F*_4,84_ = 11.9, *p* < 0.001), group x session interaction (*F*_4,84_ = 0.80, *p* = 0.53). *Post hoc* analysis indicated that the DFP-treated mice were impaired in the hidden platform task particularly on day 5 of testing compared to the vehicle-treated mice (*p* < 0.01).

#### 3.3.2 Swim speeds

Figure 2B illustrates the average swim speeds [i.e., the distance swam (cm) divided by the latency to find the platform (sec)] during water maze testing. Swim speeds averaged ~12–13 cm/s in both treatment groups and were not statistically different.

#### 3.3.3 Probe trial test

In the probe trial, the DFP-treated mice also took longer to enter a 40 cm diameter target zone centered around the previous hidden platform area (designated annulus 40-see Figure 2C) compared to controls, *p* = 0.01 (Figure 2D), while the time spent in the larger target quadrant of the previous platform location (25% of the total pool area) (Figure 2E) was not statistically different in the groups (*p* = 0.1).

### 3.4 Y-maze spontaneous alternation

**Figure 3:**
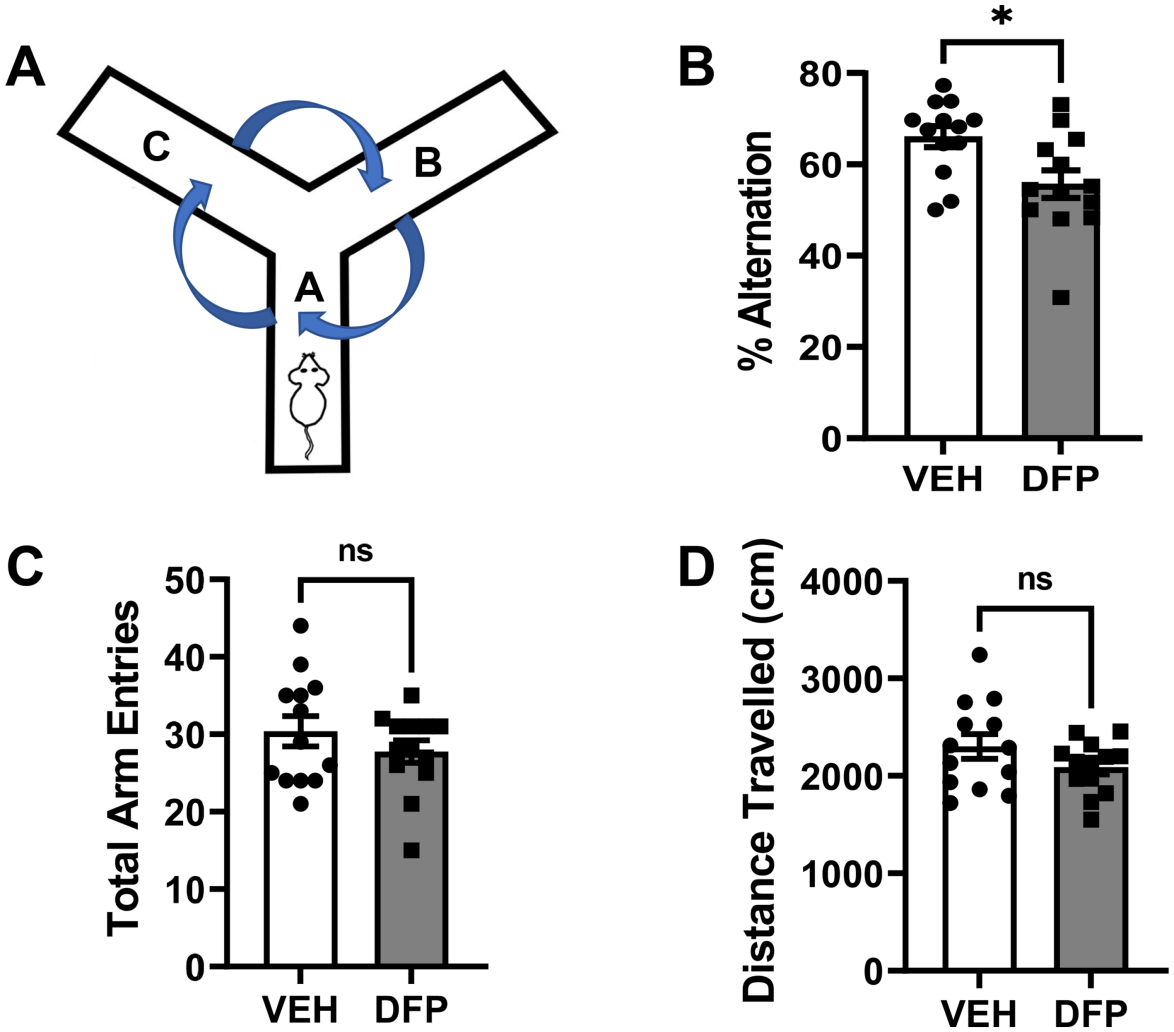
Persistent effects of DFP in a spontaneous alternation task. Beginning 14 days after a single injection of DFP 4.0 mg/kg i.p., young male mice were trained in a spontaneous alternation task for 10 min. **(A)** Diagram of the mouse Y-Maze Spontaneous Alternation Task, **(B)** % of alternations during the 10 min testing period (mean ± SEM). **(C)** Total arm entries during the 10 min testing period **(D)** Total distance traveled (cm) during the 10 min testing period (mean ± SEM) *p < 0.05, DFP- vs. vehicle-treated effect on performance. Vehicle, *N* = 11; DFP, *N* = 12.

In Figure 3, the results of studies conducted using the Y-Maze Spontaneous Alternation Task are presented. The subjects tested in this task were previously evaluated in the water maze. These experiments were initiated 14 days after DFP 4.0 mg/kg exposure and indicate that the subjects administered the OP exhibited persistent spatial working memory deficits as indicated by fewer % alternations compared to controls, *p* = 0.01 (Figure 3B) without evidence of exploratory (total arms entered, Figure 3C) or locomotor (distance traveled, Figure 3D) deficits (*p* > 0.05 for both comparisons).

### 3.5 Fear conditioning tests

#### 3.5.1 Training

**Figure 4:**
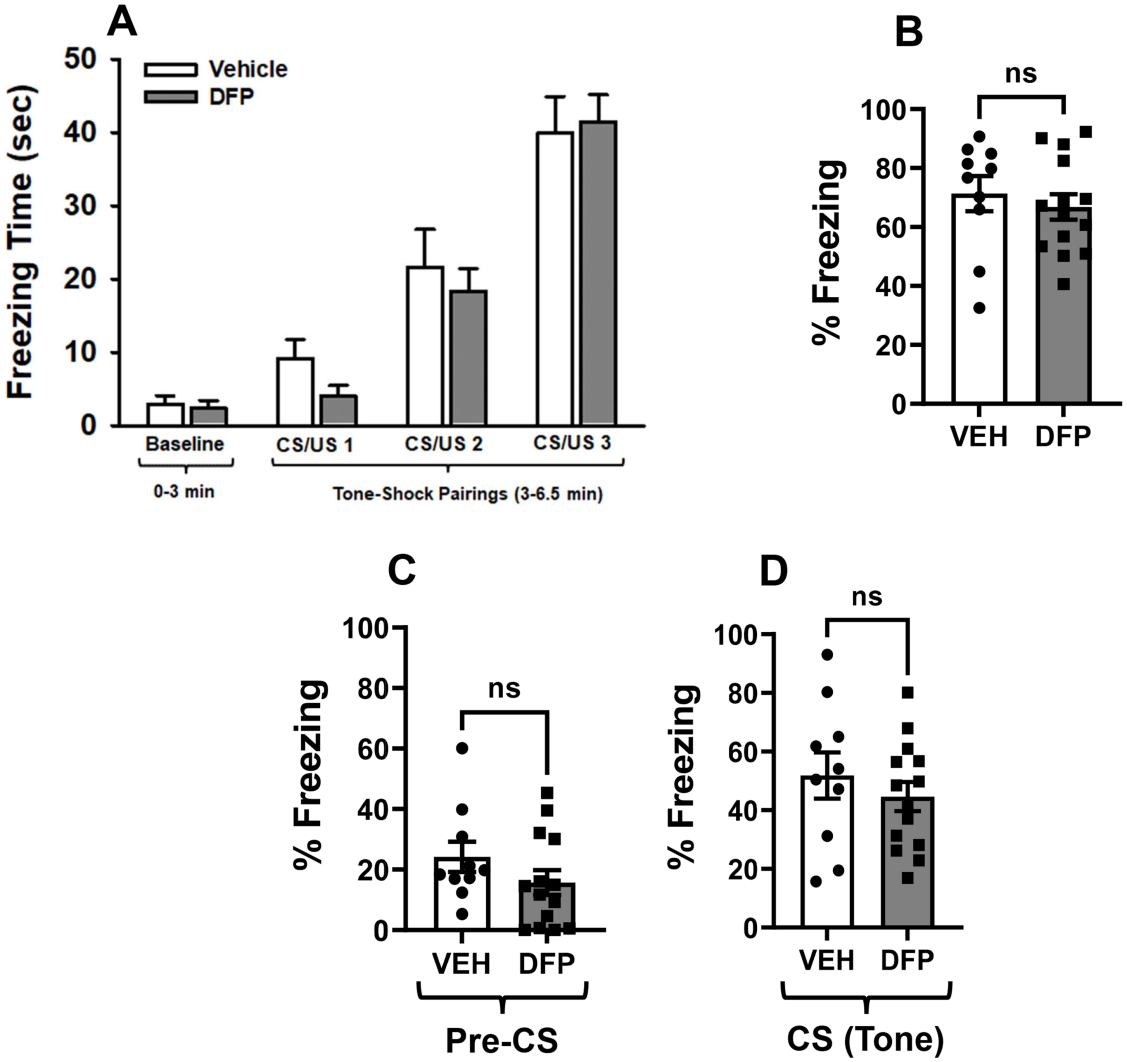
Lack of persistent effects of DFP in the fear conditioning tasks. **(A)** Mean (± S.E.M) time freezing during baseline (no shock) and after 3 tone-shock (CS/US) pairings. **(B)** Mean (± S.E.M) percentage time freezing during the eight (8) min contextual fear conditioning session conducted 24 h after the training session. **(C)** Mean (± S.E.M) percentage of time freezing during the 3 min (180 s) baseline period and **(D)** during the 5 min (300 s) tone exposure component of the session in the cued fear conditioning sessions conducted 48 h after the training session. CS = conditioning stimulus; US = unconditioned stimulus. Vehicle, *N* = 10; DFP, *N* = 14.

The time freezing in the training (conditioning) session of the Fear Conditioning Test by the two mouse groups is illustrated in Figure 4A. The time spent freezing increased in both test groups as the CS/US parings were repeated and there were no significant differences between the groups.

#### 3.5.2 Contextual fear

The % time freezing in the contextual fear conditioning sessions are illustrated in Figure 4B. During the 8 min (480 s) test session, the total times freezing averaged ~340 and ~ 320 s for the vehicle and DFP groups, respectively. There were no statistically significant group-related differences (p > 0.05) in either the total times freezing or the % of the total time freezing.

#### 3.5.3 Cued fear

The % time freezing in cued fear conditioning sessions during the 3 min (180 s) baseline period and during the 5 min (300 s) tone exposure component of the session are illustrated in Figures 4C,D respectively. The total times freezing during the baseline period (no tone) averaged ~43 and ~ 28 s for the vehicle and DFP groups, respectively. The total times freezing during the tone (cued) period averaged ~155 and ~ 134 s for the vehicle and DFP groups, respectively. There were no statistically significant group-related differences (p > 0.05) in either the total times freezing or the % of the total time freezing.

### 3.6 Assessments of visual acuity, exploratory activity, motor function, and motivation to exercise

#### 3.6.1 Visual reaching response test

All the mice in both the DFP and control groups scored a 1 (i.e., front legs stretched out as they neared the surface of the test apparatus) indicating that they had no gross visual deficits.

#### 3.6.2 Phenotyper

From the video recordings of the 18 h sessions in the Phenotyper cages, the DFP-treated subjects (compared to controls) did not show any signs of thigmotaxis (i.e., excessive time spent exploring the most peripheral parts of the cage) and there were no significant differences in the total distance traveled, number of licks on the lickometer, running wheel revolutions, time on the running wheel, number of shelter entries, or time in the shelter (see Table 2).

**Table 2:** Phenotyper data (18 hours).

		Distance	Lickometer	Running Wheel	Running Wheel	Shelter	Time in
Treatment	*N*	Traveled (cm)	# of Licks	Revolutions	Time (sec)	Entries	Shelter (sec)
Vehicle	17	23369.76 ± 2026.43	222.97 ± 30.10	1125.37 ± 141.74	1695.19 ± 176.44	42.44 ± 4.39	4166.44 ± 183.30
DFP	18	23556.03 ± 1874.20	267.77 ± 17.65	1299.17 ± 151.23	1809.90 ± 159.10	41.25 ± 3.11	3967.08 ± 180.70

All data are presented as the group means ± S.E.M.

#### 3.6.3 Grip strength, hanging wire test, rotarod

**Figure 5:**
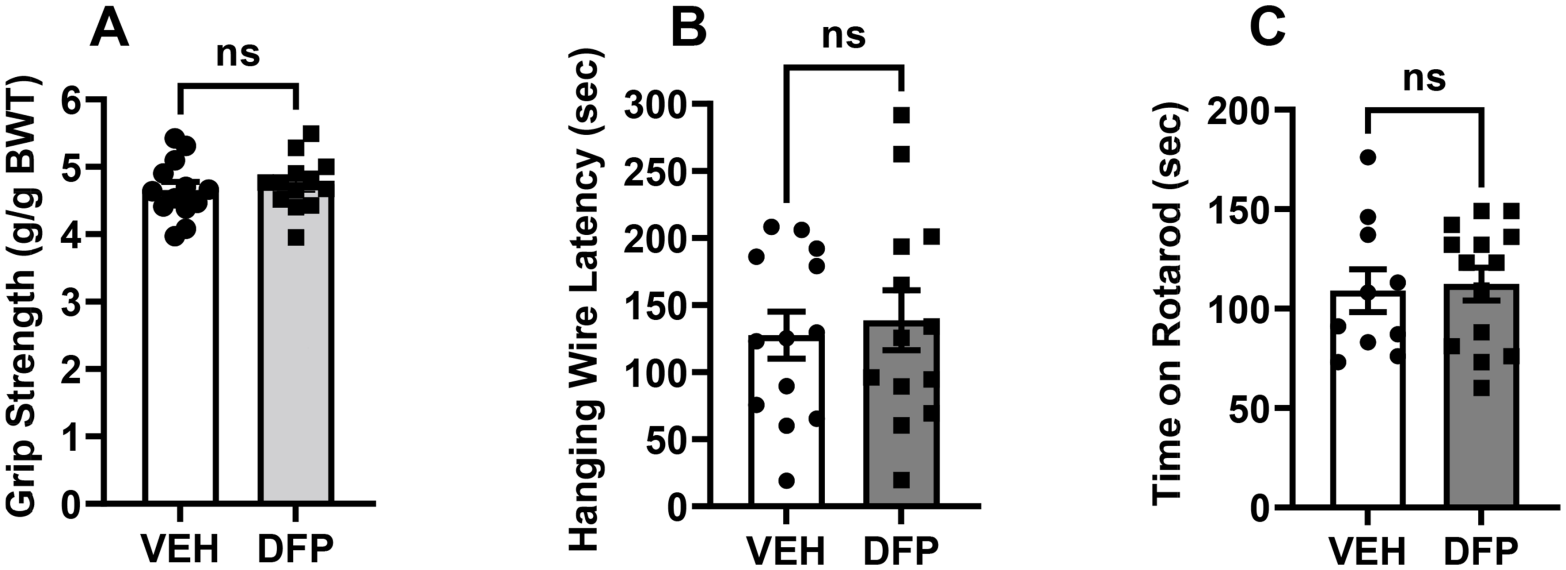
Lack of persistent effects of DFP in the non-cognitive behavioral tasks. **(A)** Mean grip strength (measured in grams of resistance ± SEM), **(B)** Mean (± S.E.M) hang time latency in sec in the Hanging Wire Test, **(C)** Mean (± S.E.M) time (sec) maintained on the accelerating rotarod. Vehicle, *N* = 11; DFP, *N* = 12.

The effects of previous DFP exposure on forelimb grip strength, latency to fall in the hanging wire test, and time on the rotarod are illustrated in Figures 5A–C, respectively. There were no significant group-related differences in any of these outcome measures.

### 3.7 Expression of cellular senescence marker p21 in the PFC of DFP-exposed mice

**Figure 6:**
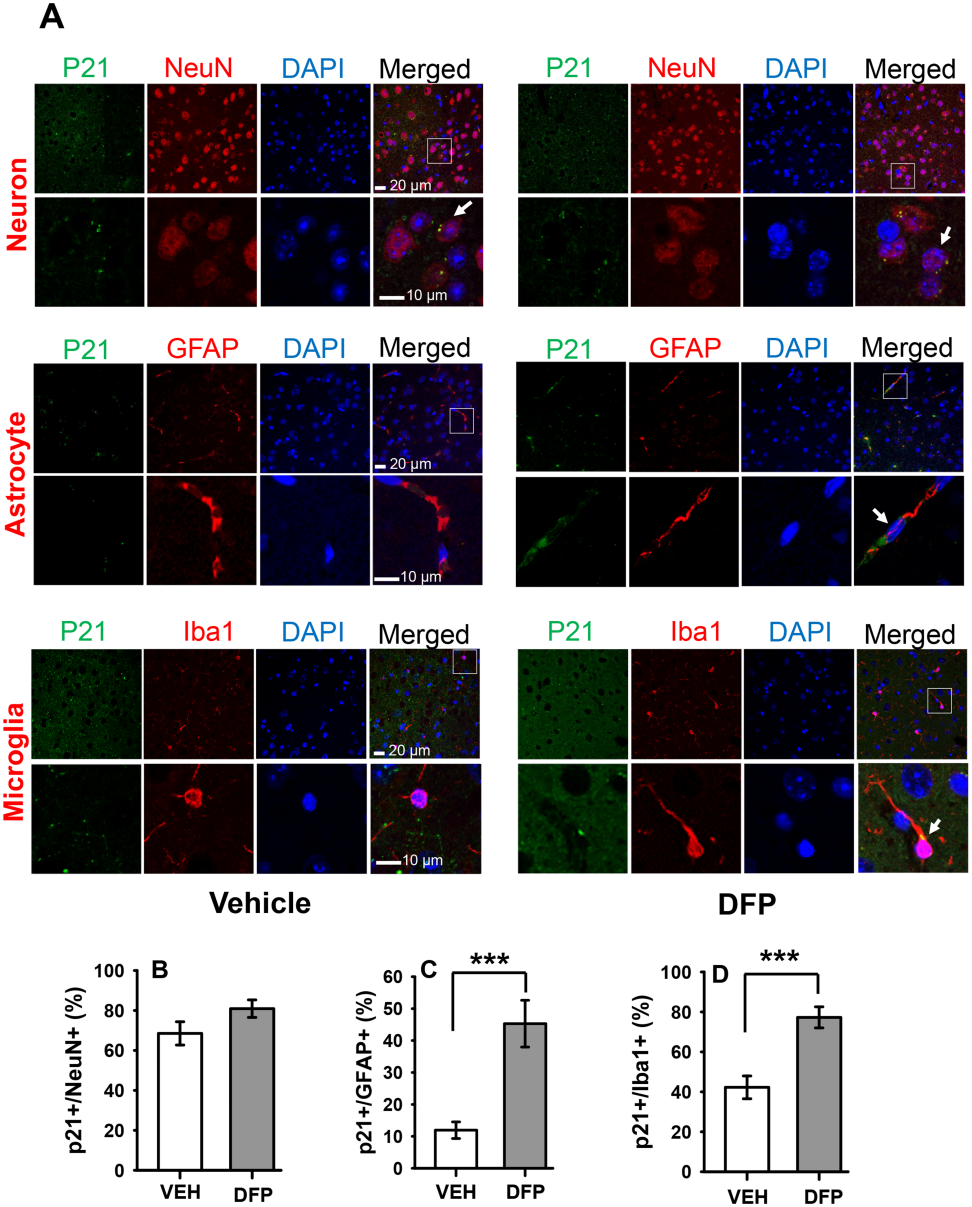
Persistent increases in the senescence marker p21 in the prefrontal cortex of mice previously exposed to DFP. The cellular senescence marker p21 was examined in the prefrontal cortex of mice 7 days after acute exposure to DFP 4.0 mg/kg **(A)** Confocal immunohistochemistry images for p21 (green), lineage-associated markers (red) for neurons (NeuN+ cells), astrocytes (GFAP + cells), microglia (Iba1 + cells), nuclear DAPI staining (blue), and the merged images. The arrows indicate co-expressing cells (p21 and lineage-associated markers). Scale bars = 10 or 20 μm. Bar plots illustrate the mean (± S.E.M) percentage of neurons **(B)**, astrocytes **(C)** and microglia **(D)** expressing the senescence marker p21 in prefrontal cortex. *N* = 3, ***p < 0.001.

To determine whether DFP exposure was associated with persistent changes in cellular senescence markers in the brain we initially performed a series of immunostaining experiments for the senescence marker p21 in slices of the PFC 7 days after exposure to DFP (i.e., in parallel with the behavioral experiments that began 6 days after DFP exposure). The results of these experiments are provided in Figure 6. The top portion of the figure (A) provides confocal images for the p21 (green) with lineage-associated markers of neurons (NeuN+ cells), astrocytes (GFAP + cells), and microglia (Iba1 + cells) (red), nuclear DAPI staining (blue), and the merged images. The Bar plots below illustrate the mean (± S.E.M) percentage of neurons (B), astrocytes (C) and microglia (D) expressing p21. Statistical analysis of the percentages of p21-labeled cells indicated that the mice exposed to DFP had a marked elevation of p21 labeling (*p* < 0.001, DFP versus controls) in astrocytes and glia, whereas in neurons, there was a non-significant trend (*p* < 0.1) toward an increase.

### 3.8 Altered expression of cellular senescence markers and proinflammatory cytokines in DFP-exposed mice

**Figure 7:**
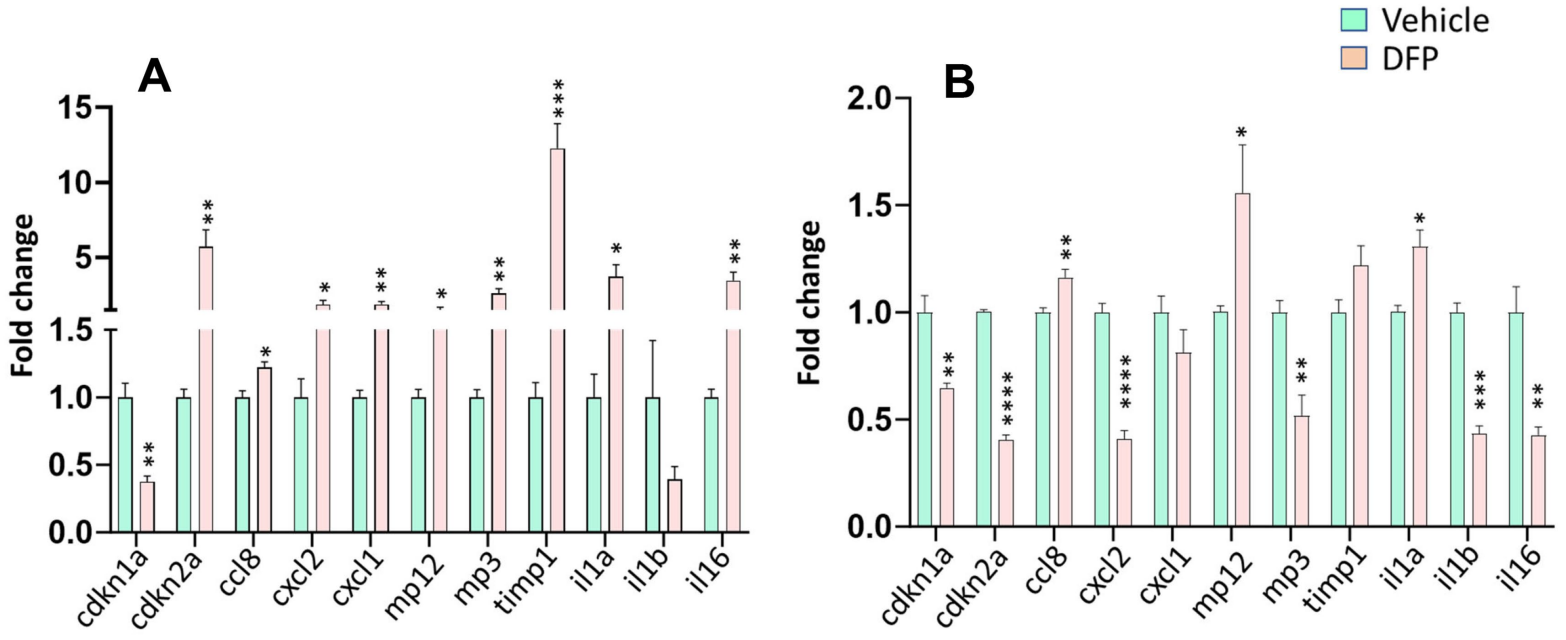
Persistent alterations in markers for senescence and proinflammatory cytokines in the brains of mice previously exposed to DFP. mRNA for markers of cellular senescence and proinflammatory cytokines were examined in the prefrontal cortex **(A)** and hippocampus **(B)** of mice previously exposed to DFP versus vehicle control by qT-PCR after a 35-day washout period. *N* = 6, *p < 0.05; **p < 0.01; ***p < 0.001; ****p < 0.0001 vs. Vehicle.

To determine whether DFP exposure was associated with more persistent changes in cellular senescence and inflammation in the brain we measured the mRNA levels of a variety of key senescence markers and cytokines that are involved in the SASP in the PFC and hippocampus at the end of the 35-day testing period (Figure 7). In the PFC, One-way ANOVA indicated significant increases in Cdkn2a, Cxcl1, Cxcl2, Ccl8, Mmp-3, Mmp-12, tmp1, il1a and il6 mRNA levels in DFP-exposed compared to control mice (Figure 7A). Surprisingly, Cdkn1a (p21) significantly decreased. In the hippocampus, like the PFC, Cdkn1a (p21) was significantly decreased and Ccl8, Mmp-12, il1a were increased and there was a nonsignificant trend (*p* > 0.1) toward an increase in tmp1 (Figure 7B). However, in contrast to the PFC, Cdkn2a, Cxcl2, Mmp-3, il1b and il6 were significantly decreased.

## 4 Discussion

The major findings of this rodent study can be summarized as follows: (1) previous exposure to a 4.0 mg/kg dose of DFP in mice was associated with modest, but persistent impairments in both acquisition and recall in a water maze spatial learning task as well as working memory deficits in a spontaneous alternation version of the Y-Maze task; (2) previous DFP exposure was not associated with negative effects in either a contextual or cued fear conditioning test, (3) previous DFP exposure was not associated with negative effects on weight or impairments in any of the various noncognitive behavioral assessments including the visual reaching response test, Phenotyper assessment, grip strength, hanging wire, rotarod, or swim speeds in the water maze, (4) both histology experiments and quantitative PCR experiments indicated that previous exposure to DFP was associated with alterations in several senescence markers and proinflammatory cytokines in brain regions that are relevant to the performance of the memory-related tasks (e.g., hippocampus, PFC).

In behavioral experiments, the water maze procedure was utilized since it is a visuospatial learning task that is sensitive cholinergic alterations (e.g., McNamara and Skelton, 1993), and importantly, deficits in visuospatial processing have been identified as one of the negative outcomes in patients previously exposed to OPs (Roldán-Tapia et al., 2005). The water maze (spatial learning and recall) procedure was employed in this study since it also requires the hippocampus, important components of human learning and memory such as information acquisition and encoding, consolidation, retention, and retrieval (McNamara and Skelton, 1993; McDonald and White, 1995). In the present study, mice previously exposed to DFP were impaired in the hidden platform task as well as in probe trials indicating persistent (DFP-related) disruptions in spatial reference learning (acquisition) as well as retention (recall).

As an adjunct to the water maze studies, we also conducted a spatial working memory task, Y-maze Spontaneous Alternation and well as both contextual and cued fear conditioning tasks. Y-maze tasks are based on the innate tendency of rodents to explore novel environments (Dellu et al., 2000) and they are simple to perform and do not require rule learning, extensive handling, or manipulations (Heredia-López et al., 2016) or exposure to aversive conditions (e.g., food restriction, electrical shock). The free exploration version of the task, Spontaneous Alternation, is dependent on the PFC and the hippocampus and other relevant brain regions (basal forebrain), as well as cholinergic function (Lalonde, 2002). In the present study, mice previously exposed to DFP exhibited impairments in Spontaneous Alternation, a working memory-related behavior. Contextual and cued fear-conditioning tasks measure memory of an aversive experience and the stimuli present during this aversive experience. Various types of instruments are used to control a foot shock delivery and to measure the duration of freezing behavior which is the standard response (dependent measure) to this sudden aversive stimulus. Freezing is defined as a complete behavioral immobility except for natural respiratory motions. Reduced freezing in the context and cue tests has been observed in rodents with amygdala lesions and has been argued to represent diminished fear, while the reduction of freezing only in the context test has been suggested to be a sign of hippocampal dependent (contextual) memory impairment (Phillips and LeDoux, 1992). In the Contextual Fear conditioning task conducted 24 h after the training session in this study, all the subjects demonstrated recall (freezing) of the contextual cues and there were no significant group-related differences. Likewise, in the Cued Fear Conditioning task conducted 48 h after the training session all of the subjects demonstrated recall (freezing) of the auditory cue (tone) and there were no significant group-related differences.

The results of the cognitive-behavioral tasks described above thus indicate that in this particular OP dosing model (acute, relatively high dose DFP exposure), persistent, cognitive domain specific, impairments were observed (i.e., persistent deficits in spatial learning and recall and working memory, but not contextual or cue-related fear memory). The observations in the non-cognitive behavioral assessments described above argue against the premise that persistent DFP-related effects on general health status or on motor function, fatigue, motivation to exercise, visual acuity, exploratory activity, anhedonia, or anxiety-related behaviors could explain the deficits in performance of the memory-related behavioral tasks.

To determine whether DFP exposure was associated with persistent changes in cellular senescence in the brain during the same time period as when behavioral experiments were initiated, we initially performed a series of immunostaining experiments for the senescence marker p21 in slices of the PFC 7 days after exposure to DFP. Senescent cells exhibit marked alterations in gene expression, and p21 (CDKN1a) is one of two commonly measured senescence markers along with p16 (CDKN2a). These cell-cycle inhibitors are cyclin-dependent kinase inhibitors (CDKIs) and components of tumor-suppressor pathways that can establish and maintain the growth arrest that is typical of senescence (see Campisi and D’Adda di Fagagna, 2007). We analyzed astrocytes, microglia and neurons in these experiments. Observations of age-related senescence in astrocytes and microglia have led to the hypothesis that a disruption of glia–neuron interactions through the SASP may lead to the development of age-related brain pathologies. While the induction of senescence and a SASP in post-mitotic cells is less well understood, there is some evidence of these processes occurring in neurons of ageing mice suggesting that these processes might not be restricted to proliferating cells (see Vazquez-Villaseñor et al., 2020). In our studies, we observed robust increases in p21 after DFP exposure in both astrocytes and microglia, but only a small (non-statistically significant) increase in neurons.

These initial histological observations led us to perform the q-RT-PCR experiments at the end of the 35-day test period to determine if more persistent effects DFP exposure might be observed. While not cell type-specific, these experiments allowed us to perform a screen of mRNAs across multiple senescence markers and proinflammatory cytokines that are involved in the SASP. Surprisingly, the mRNA for p21 (Cdkn1) was significantly decreased in both PFC and hippocampus at this 35-day post DFP exposure, essentially the opposite effects from what was observed in the histology experiments at 7 days post OP exposure in the PFC. In the PFC we observed quite robust increases in p16 (Cdkn2a), Cxcl1, Cxcl2, Ccl8, Mmp-3, Mmp-12, tmp1, il1a and il6 mRNA levels. In the hippocampus, like the PFC, Ccl8, Mmp-12, and il1a were increased, while there was a nonsignificant trend (*p* > 0.1) toward an increase in tmp1. However, in contrast to the PFC, Cdkn2a, Cxcl2, Mmp-3, il1b and il6 were significantly decreased. These results suggest a reciprocal interaction between the PFC and hippocampus in the regulation of senescence-mediated pathways after DFP exposure. Also, these changes could reflect the differential expression pattern of senescence markers in different neural cell types in these two brain regions as we observed in our immunofluorescence data from the PFC tissues where a difference in the expression of p21 in neurons vs. non-neuronal cell types following DFP exposure was present. Additional studies to examine the cell-type specific expression of senescence markers in both PFC and hippocampus are warranted to understand the basis for the brain region specific alterations in senescence markers.

It should be noted that while the rationale for our experiments was to determine if acute exposure to an OP might lead to long-term elevations in markers for senescence and inflammaging in the brain, it is possible that a single OP exposure and the relatively young age of the mice at the time of exposure (3 months old) resulted in temporary alterations in homeostatic processes leading to increases or decreases in senescence markers and proinflammatory cytokines that would resolve over time. In the previously published studies that we cited as providing the impetus for our studies using the single 4.0 mg/kg dose of DFP, the mRNAs for several proinflammatory cytokines and chemokines were increased in multiple brain regions (including frontal cortex and hippocampus) (O’Callaghan et al., 2015; Locker et al., 2017) and some of the same mRNAs we analyzed (e.g., il16, il1b), were increased. However, the increases were observed at 2 h post DFP, increasing further by 6 h, but reducing toward control levels by 12 h post DFP.

It should also be noted, however, that a recent study in adult male Sprague–Dawley rats administered DFP (4 mg/kg, s.c.) then subsequently injected with antidotal rescue agents, the antimuscarinic, atropine sulfate and the AChE reactivating agent, 2-pralidoxime (25 mg/kg, i.m.) showed both a time dependent and neuron-specific appearance of senescence markers after drug exposure (Tsai et al., 2024). Specifically, in immunohistochemical studies, co-localization of p16 immunoreactivity with cell-specific biomarkers showed no cellular senescence at 1-month post-exposure, however, at 3- and 6-months post-exposure, p16 immunoreactivity was significantly increased in a neuron specific manner in several brain regions including the CA3 and dentate gyrus of the hippocampus, amygdala, piriform cortex and thalamus, but not the CA1 region of the hippocampus or the somatosensory cortex. While our DFP study in mice without rescue agents are not be directly comparable to the studies described above, both studies indicate persistent cellular senescence associated with OP exposure and the latter studies suggest that the effects may become further exacerbated as the exposed subject ages.

On first glance, a causal link between OP exposures and the chronic illnesses described in the Introduction above, as well as, potentially, premature aging observed in Gulf War veterans, seems plausible since both acutely toxic and repeated subacute exposures to OPs in animals have been shown to result in sustained increases reactive oxygen species (ROS) and oxidative stress, mitochondrial dysfunction, lipid peroxidation and neuroinflammation (reviewed, Guignet and Lein, 2019; Tsai and Lein, 2021; i.e., toxicological effects that mirror several neuropathological features of advanced biological aging, PD, and AD). The conclusions of the epidemiological studies linking OP exposures to age-related neurodegenerative illnesses have been debated, however, and some published reviews point to inconsistencies in this literature (see Baltazar et al., 2014; Sánchez-Santed et al., 2016). Moreover, the interpretation of epidemiological findings can be complicated by the complexity of chemical exposures, poor exposure records, problems with bias and recall in self-reporting, etc. (reviewed in Voorhees et al., 2017). Thus, controlled studies in preclinical models such as those described here are essential for establishing cause-effect relationships between OP exposures and adverse long-term health outcomes that are believed to occur in humans.

There are some limitations to the studies described here that we plan to address in future experiments. In these initial studies, we only used male mice to obviate the challenges associated with estrous cycles in females. Future studies will include both male and female mice to determine if there are sex specific differences in senescence associated with OP-exposure. We will also evaluate additional brain regions beyond PFC and the hippocampus as well as subfields of the hippocampus similar to the aforementioned (Tsai et al., 2024) rat study. Moreover, we will move beyond histology assessments and q-RT-PCR experiments and confirm changes in the levels of senescence markers in specific brain regions with immunoblotting of several specific senescence markers including p16, p21, p53, γH.2AX, and SA-*β*-gal.

In conclusion, the studies described here in mice indicate that acute relatively high dose DFP exposure can lead to persistent impairments in some domains of cognition as well as persistent alterations in markers for cellular senescence and inflammaging.

## Data Availability

The raw data supporting the conclusions of this article will be made available by the authors, without undue reservation.
