# Supplementary material for: Acute exposure to diisopropylfluorophosphate in mice results in persistent cognitive deficits and alterations in senescence markers in the brain
Source: Front Neurosci. 2024 Nov 7;18:1498350. doi: 10.3389/fnins.2024.1498350 (PMC11578986; doi:10.3389/fnins.2024.1498350)
Supplement: Supplementary file 1 [file Table_1.docx]

**Table-1: Primer list**

| **Name** | **Sequence** |
| --- | --- |
| Cdkn1a -F | GCAGATCCACAGCGATATCCA |
| Cdkn1a R | AACAGGTCGGACATCACCAG |
| Cdkn2a-F | CCCAACGCCCCGAACT |
| Cdkn2a-R | GCAGAAGAGCTGCTACGTGAA |
| IL6-F | TGAGAAAAGAGTTGTGCAATGG |
| IL6-R | GGTACTCCAGAAGACCAGAGG |
| Il1a-F | AGGGAGTCAACTCATTGGCG |
| Il1a-R | TGGCAGAACTGTAGTCTTCGT |
| Il1b-F | TGCCACCTTTTGACAGTGATG |
| Il1b-R | TGATGTGCTGCTGCGAGATT |
| Timp1-F | CACACCAGAGCAGATACCATGA |
| Timp1-R | GGGGAACCCATGAATTTAGCC |
| Mmp3-F | GTTGGAGAACATGGAGACTTTGT |
| Mmp3-R | CAAGTTCATGAGCAGCAACCA |
| Mmp12-F | TGCACTCTGCTGAAAGGAGTCT |
| Mmp12-R | GTCATTGGAATTCTGTCCTTTCCA |
| Cxcl1-F | ACCGAAGTCATAGCCACACTC |
| Cxcl1-R | CTCCGTTACTTGGGGACACC |
| Cxcl2-F | CCCAGACAGAAGTCATAGCCAC |
| Cxcl2-R | TGGTTCTTCCGTTGAGGGAC |
| Ccl8-F | CGGGTGCTGAAAAGCTACGA |
| Ccl8-R | TTGGTCTGGAAAACCACAGCTT |
| Actb-F | GTCCACACCCGCCACC |
| Actb-R | ACCCATTCCCACCATCACAC |
| B2M-F | CCCCACTGAGACTGATACATACG |
| B2M-R | CGATCCCAGTAGACGGTCTTG |
